# Poly[(μ-2,3-diethyl-7,8-di­methyl­quinoxaline-κ^2^ *N*:*N*)(2,3-diethyl-7,8-di­methyl­quinoxaline-κ*N*)-μ-nitrato-κ^2^ *O*:*O*′-nitrato-κ^2^ *O*,*O*′-disilver(I)]

**DOI:** 10.1107/S2414314624002475

**Published:** 2024-03-21

**Authors:** Guy Crundwell, Ashley Leeds

**Affiliations:** a Central Connecticut State University, Department of Chemistry & Biochemistry, 1619 Stanley Street, New Britain, CT 06053, USA; Purdue University, USA

**Keywords:** crystal structure, silver, quinoxaline, extended network

## Abstract

The structure of the title compound, [C_14_H_18_N_2_)_2_Ag_2_](NO_3_)_2_, contains subtle differences in ligand, metal, and counter-anion coordination. One quinoxaline ligand uses one of its quinoxaline N atoms to bond to one silver cation. That silver cation is bound to a second quinoxaline which, in turn, is bound to a second silver atom; thereby using both of its quinoxaline N atoms. A nitrate group bonds with one of its O atoms to the first silver and uses the same oxygen to bond to a silver atom (related by symmetry to the second), thereby forming an extended network. The second nitrate group on the other silver bonds *via* two nitrate O atoms; one silver cation therefore has a coordination number of three whereas the second has a coordination number of four. One of the quinoxaline ligands has a disordered ethyl group.

## Abstract 2

The structure of the title compound, [Ag_2_(NO_3_)_2_(C_14_H_18_N_2_)_2_]_*n*_, contains subtle differences in ligand, metal, and counter-anion coordination. One quinoxaline ligand uses one of its quinoxaline N atoms to bond to one silver cation. That silver cation is bound to a second quinoxaline which, in turn, is bound to a second silver atom, thereby using both of its quinoxaline N atoms. A nitrate group bonds with one of its O atoms to the first silver and uses the same oxygen to bond to a silver atom (related by symmetry to the second), thereby forming an extended network. The second nitrate group on the other silver bonds *via* two nitrate O atoms; one silver cation therefore has a coordination number of three whereas the second has a coordination number of four.

## Graphical abstract

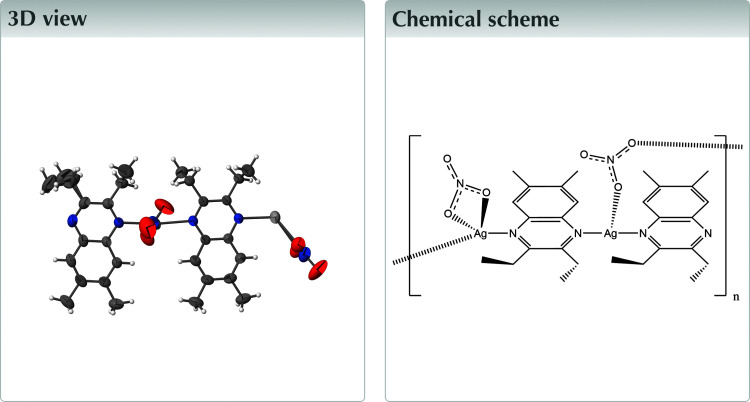

## Structure description

There are many known structures of polymeric silver(I) quinoxaline complexes. Yeh *et al.* (2009 ▸) have made *catena* complexes of silver and 2,3-di­phenyl­quinoxaline with tetra­fluoro­borate in water, tetra­fluoro­borate in aceto­nitrile, perchlorate in aceto­nitrile, tri­fluoro­methane­sulfonate, and hexa­fluoro­anti­monate salts. When they used nitrate salts, be they in water, di­methyl­formamide, or aceto­nitrile, the nitrate counter-anions acted as bridging ligands; in addition, in all of the structures, regardless of solvent or counter-anion, the quinoxaline ligands are always bidentate and bridge silver cations. Patra *et al.* (2007 ▸) also studied several *catena* complexes of 1:1 molar amounts of silver with 2,3-di­phenyl­quinoxaline–silver perchlorate from methanol, silver tetra­fluoro­borate from ethanol, and again with silver nitrate to name a few. In all of these structures, the quinoxaline is bidentate and bridging and nitrate ions (if present) bridge silver cations. Finally, cationic silver–di­phenyl­quinoxaline polymeric networks can even be isolated with large phosphato–molybdenum oxide anion clusters (Tian *et al.*, 2016 ▸). As with the other complexes, the quinoxalines are bidentate and bridge silver cations.

**Figure 1:**
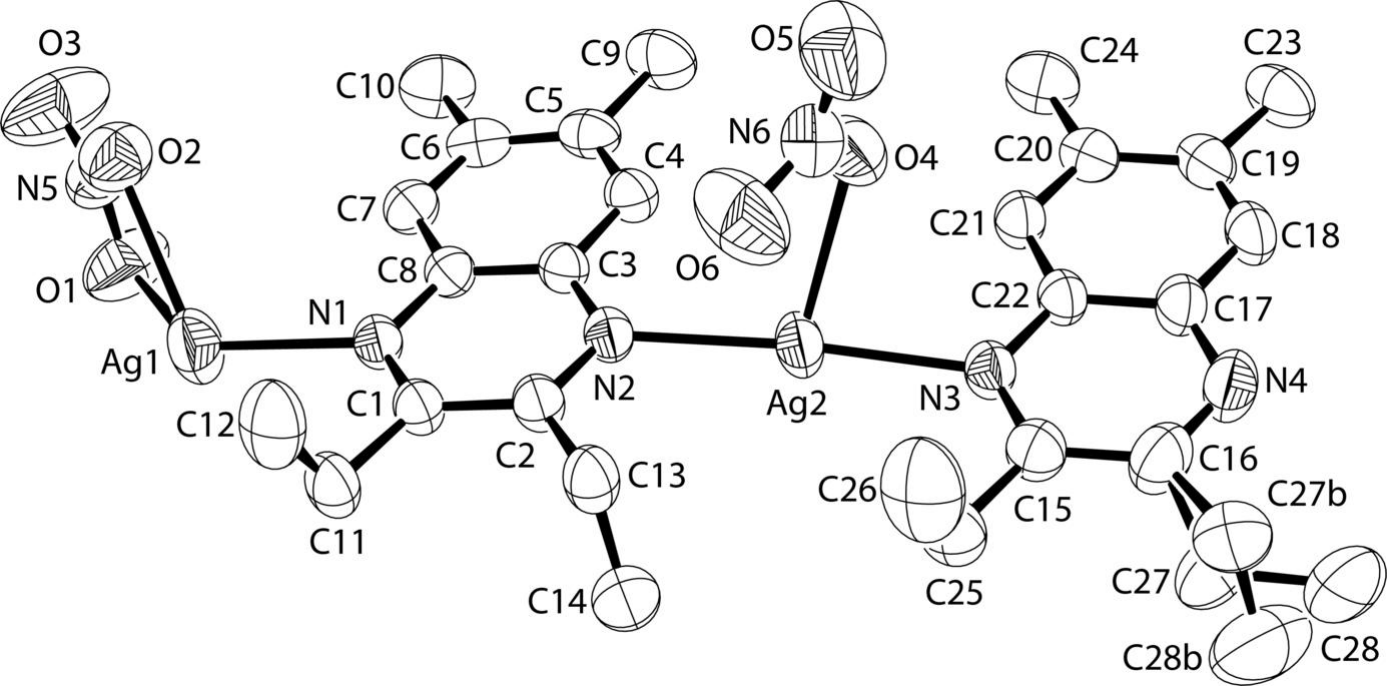
A view of the title compound (Farrugia, 2012 ▸). Displacement ellipsoids are drawn at the 50% probability level.

This is the first structure of a silver *catena* complex with 2,3-diethyl-7,8-di­methyl­quinoxaline; however, unlike previous structures, the bonding behavior of the quinoxaline ligand is varied. There are subtle differences in ligand, metal, and counter-anion coordination in the crystal. The structure can be described loosely as a dimer – two sets of a metal, a ligand, and an anion; however, each part of those two sets has inter­esting differences. As can be seen in Fig. 1 ▸, the first silver atom (Ag1) is bound to a bidentate nitrate anion [with Ag—O distances of 2.498 (2) Å and 2.512 (2) Å] and a quinoxaline nitro­gen (N1) at 2.2600 (17) Å. What is not seen in the *ORTEP* is that the silver is also bound to a bridging oxygen from the second nitrate (O4) at 2.3195 (19) Å, making the silver four-coord­inate. The first quinoxaline (on the left in Fig. 1 ▸) is bidentate and bridging; making a bond with the second silver (Ag2) at 2.2492 (17) Å. The di­methyl­quinoxaline portion of the ligand is essentially flat, whereas the ethyl groups dangle above and below the plane formed by the dimer. The second silver (Ag2) is three-coordinate and bridges the two quinoxalines [Ag2—N3 has a bond distance of 2.2552 (17) Å], while also being bound to a bridging nitrate anion oxygen at a distance of 2.5956 (19) Å. The N2—Ag2—N3 bond angle is essentially linear at 173.50 (6)° which is commonly seen in bis- and *catena* complexes of silver(I). Finally, the dimer is capped by a second 2,3-diethyl-7,8-di­methyl­quinoxaline ligand. This ligand is monodentate and is not bridging. Also, unlike the other ligand, this quinoxaline exhibits a positional disorder of its outer ethyl group. The disordered ethyl group was refined to be 59.6 (1)/40.4 (1)%.

## Synthesis and crystallization

Silver nitrate was used as received from Fisher Scientific. The ligand, 2,3-diethyl-7,8-di­methyl­quinoxaline, was synthesized from the condensation of 4,5-dimethyl-1,2-phenyl­enedi­amine with 3,4-hexa­nedione. Purity of the ligand was confirmed prior to use by ^1^H NMR. A 30 ml solution of 43 mg (0.20 mmol) of 2,3-diethyl-7,8-di­methyl­quinoxaline in warmed methanol was combined with a 10 ml methanol solution of 34 g (0.20 mmol) of silver nitrate and stirred for 1 minute. The solution was taken off heat and pipetted into test tubes which were covered with parafilm and place in amber vials in a drawer to keep them from direct light. Diffraction-quality, colorless crystals formed *via* slow evaporation of the solvent within 48–72 h. Crystals were harvested from the evaporating solutions and used immediately due to the decay of the silver(I) complex in light.

## Refinement

Crystal data, data collection and structure refinement details are summarized in Table 1 ▸. One of the ethyl groups in a 2,3-diethyl-7,8-di­methyl­quinoxaline are disordered. The thermal displacement parameters of the disordered carbons in the group were restrained as the amount of disorder was refined. The percent disorder of the ethyl group was determined to be 59.6 (1)/40.4 (1)%. Thermal displacement parameters for the nitrate atoms were also restrained during refinement.

## Supplementary Material

Crystal structure: contains datablock(s) I. DOI: 10.1107/S2414314624002475/zl4069sup1.cif

Structure factors: contains datablock(s) I. DOI: 10.1107/S2414314624002475/zl4069Isup2.hkl

CCDC reference: 2340469

Additional supporting information:  crystallographic information; 3D view; checkCIF report

## Figures and Tables

**Table 1:** Experimental details

Crystal data	
Chemical formula	[Ag_2_(NO_3_)_2_(C_14_H_18_N_2_)_2_]
*M*_r_	768.37
Crystal system, space group	Monoclinic, *P*2_1_/*n*
Temperature (K)	293
*a*, *b*, *c* (Å)	10.3048 (2), 24.1140 (6), 12.6416 (4)
β (°)	100.911 (3)
*V* (Å^3^)	3084.53 (13)
*Z*	4
Radiation type	Mo *K*α
μ (mm^−1^)	1.32
Crystal size (mm)	0.41 × 0.33 × 0.25

Data collection	
Diffractometer	Xcalibur, Sapphire3
Absorption correction	Multi-scan (*CrysAlis PRO*; Rigaku OD, 2019 ▸)
*T*_min_, *T*_max_	0.775, 1.000
No. of measured, independent and observed [*I* > 2σ(*I*)] reflections	36657, 11225, 6825
*R*_int_	0.030
(sin θ/λ)_max_ (Å^−1^)	0.778

Refinement	
*R*[*F* ^2^ > 2σ(*F* ^2^)], *wR*(*F* ^2^), *S*	0.039, 0.096, 1.00
No. of reflections	11225
No. of parameters	407
No. of restraints	102
H-atom treatment	H-atom parameters constrained
Δρ_max_, Δρ_min_ (e Å^−3^)	0.36, −0.49

Computer programs: *CrysAlis PRO* (Rigaku OD, 2019 ▸), *SHELXS97* (Sheldrick, 2008 ▸), *SHELXL2019/3* (Sheldrick, 2015 ▸) and *OLEX2* (Dolomanov *et al.*, 2009 ▸).

## full crystallographic data

### Crystal data

**Table:**

[Ag_2_(NO_3_)_2_(C_14_H_18_N_2_)_2_]	*F*(000) = 1552
*M**_r_* = 768.37	*D*_x_ = 1.655 Mg m^−^^3^
Monoclinic, *P*2_1_/*n*	Mo *K*α radiation, λ = 0.71073 Å
*a* = 10.3048 (2) Å	Cell parameters from 7239 reflections
*b* = 24.1140 (6) Å	θ = 3.2–31.2°
*c* = 12.6416 (4) Å	µ = 1.32 mm^−^^1^
β = 100.911 (3)°	*T* = 293 K
*V* = 3084.53 (13) Å^3^	Block, colorless
*Z* = 4	0.41 × 0.33 × 0.25 mm

### Data collection

**Table:**

Xcalibur, Sapphire3 diffractometer	11225 independent reflections
Radiation source: fine-focus sealed X-ray tube, Enhance (Mo) X-ray Source	6825 reflections with *I* > 2σ(*I*)
Graphite monochromator	*R*_int_ = 0.030
Detector resolution: 16.1790 pixels mm^-1^	θ_max_ = 33.6°, θ_min_ = 2.9°
ω scans	*h* = −12→16
Absorption correction: multi-scan (CrysAlisPro; Rigaku OD, 2019)	*k* = −36→37
*T*_min_ = 0.775, *T*_max_ = 1.000	*l* = −19→19
36657 measured reflections	

### Refinement

**Table:**

Refinement on *F*^2^	Primary atom site location: structure-invariant direct methods
Least-squares matrix: full	Hydrogen site location: inferred from neighbouring sites
*R*[*F*^2^ > 2σ(*F*^2^)] = 0.039	H-atom parameters constrained
*wR*(*F*^2^) = 0.096	*w* = 1/[σ^2^(*F*_o_^2^) + (0.0359*P*)^2^ + 0.7322*P*] where *P* = (*F*_o_^2^ + 2*F*_c_^2^)/3
*S* = 1.00	(Δ/σ)_max_ = 0.001
11225 reflections	Δρ_max_ = 0.36 e Å^−^^3^
407 parameters	Δρ_min_ = −0.49 e Å^−^^3^
102 restraints	

### Special details

**Table:**

Geometry. All esds (except the esd in the dihedral angle between two l.s. planes) are estimated using the full covariance matrix. The cell esds are taken into account individually in the estimation of esds in distances, angles and torsion angles; correlations between esds in cell parameters are only used when they are defined by crystal symmetry. An approximate (isotropic) treatment of cell esds is used for estimating esds involving l.s. planes.
Refinement. Hydrogen atoms on *sp*^2^ and *sp*^3^ carbons were placed at calculated positions with a C—H distance of 0.93 Å and 0.96 Å and were included in the refinement in riding motion approximation with *U*_iso_ = 1.2*U*_eq_ or 1.5*U*_eq_ of the carrier atom, respectively.

### Fractional atomic coordinates and isotropic or equivalent isotropic displacement parameters (Å^2^)

**Table:**

	*x*	*y*	*z*	*U*_iso_*/*U*_eq_	Occ. (<1)
Ag1	−0.14525 (2)	0.20877 (2)	0.97995 (2)	0.07499 (8)	
Ag2	0.46103 (2)	0.14066 (2)	0.79131 (2)	0.05543 (7)	
O1	−0.2455 (3)	0.30344 (10)	0.96797 (18)	0.0987 (8)	
O2	−0.1200 (2)	0.28503 (9)	1.11773 (16)	0.0775 (6)	
O3	−0.2180 (3)	0.36261 (12)	1.0993 (2)	0.1337 (12)	
O4	0.65506 (19)	0.16154 (10)	0.94950 (16)	0.0773 (6)	
O5	0.7486 (3)	0.13377 (12)	1.1028 (2)	0.1105 (9)	
O6	0.5601 (3)	0.09973 (12)	1.0286 (2)	0.1210 (9)	
N1	0.04723 (17)	0.18545 (7)	0.93064 (14)	0.0420 (4)	
N2	0.28512 (17)	0.15893 (7)	0.86719 (14)	0.0432 (4)	
N3	0.63183 (17)	0.11208 (7)	0.71683 (14)	0.0456 (4)	
N4	0.8581 (2)	0.07533 (9)	0.64542 (19)	0.0641 (5)	
N5	−0.1946 (3)	0.31748 (11)	1.0619 (2)	0.0733 (6)	
N6	0.6550 (2)	0.12997 (11)	1.0286 (2)	0.0679 (6)	
C1	0.0822 (2)	0.13332 (8)	0.92080 (17)	0.0427 (4)	
C2	0.2046 (2)	0.11947 (9)	0.88956 (18)	0.0446 (5)	
C3	0.2505 (2)	0.21320 (8)	0.87823 (16)	0.0395 (4)	
C4	0.3359 (2)	0.25626 (9)	0.85985 (17)	0.0488 (5)	
H4	0.416424	0.247454	0.841048	0.059*	
C5	0.3027 (2)	0.31103 (9)	0.86910 (17)	0.0502 (5)	
C6	0.1787 (2)	0.32432 (9)	0.89600 (17)	0.0507 (5)	
C7	0.0972 (2)	0.28286 (9)	0.91814 (17)	0.0472 (5)	
H7	0.018040	0.291969	0.939103	0.057*	
C8	0.1309 (2)	0.22665 (8)	0.90976 (16)	0.0397 (4)	
C9	0.3989 (3)	0.35595 (11)	0.8517 (2)	0.0707 (8)	
H9A	0.481081	0.339371	0.843480	0.106*	
H9B	0.413660	0.380471	0.912574	0.106*	
H9C	0.362848	0.376525	0.787826	0.106*	
C10	0.1338 (3)	0.38397 (10)	0.8971 (3)	0.0752 (8)	
H10A	0.201248	0.405602	0.941353	0.113*	
H10B	0.053914	0.385828	0.925588	0.113*	
H10C	0.117678	0.398347	0.824958	0.113*	
C11	−0.0104 (2)	0.08953 (10)	0.9478 (2)	0.0578 (6)	
H11A	−0.002809	0.056545	0.905528	0.069*	
H11B	−0.100595	0.102873	0.928672	0.069*	
C12	0.0193 (3)	0.07459 (12)	1.0669 (2)	0.0775 (8)	
H12A	−0.044023	0.047837	1.081579	0.116*	
H12B	0.014014	0.107320	1.109146	0.116*	
H12C	0.106552	0.059182	1.085205	0.116*	
C13	0.2465 (2)	0.06016 (9)	0.8794 (2)	0.0554 (6)	
H13A	0.216330	0.038035	0.934057	0.066*	
H13B	0.342299	0.058425	0.892742	0.066*	
C14	0.1930 (3)	0.03547 (11)	0.7699 (2)	0.0715 (7)	
H14A	0.225652	0.056205	0.715590	0.107*	
H14B	0.098237	0.036948	0.756165	0.107*	
H14C	0.221257	−0.002413	0.768422	0.107*	
C15	0.6497 (2)	0.05903 (10)	0.6971 (2)	0.0540 (5)	
C16	0.7649 (3)	0.04099 (11)	0.6597 (2)	0.0690 (7)	
C17	0.8409 (2)	0.13033 (10)	0.66478 (18)	0.0502 (5)	
C18	0.9360 (2)	0.16915 (11)	0.64532 (19)	0.0557 (6)	
H18	1.012429	0.156529	0.624060	0.067*	
C19	0.9188 (2)	0.22453 (11)	0.65687 (17)	0.0532 (6)	
C20	0.8031 (2)	0.24404 (9)	0.69231 (17)	0.0485 (5)	
C21	0.7112 (2)	0.20651 (9)	0.71427 (18)	0.0476 (5)	
H21	0.637117	0.219201	0.739108	0.057*	
C22	0.7273 (2)	0.14926 (9)	0.69975 (17)	0.0430 (5)	
C23	1.0185 (3)	0.26514 (13)	0.6288 (2)	0.0705 (8)	
H23A	1.090685	0.245182	0.608650	0.106*	
H23B	1.051032	0.288095	0.690112	0.106*	
H23C	0.977148	0.287968	0.569713	0.106*	
C24	0.7797 (3)	0.30509 (11)	0.7034 (2)	0.0699 (7)	
H24A	0.849340	0.320529	0.756530	0.105*	
H24B	0.696477	0.310717	0.725358	0.105*	
H24C	0.778444	0.323040	0.635432	0.105*	
C25	0.5457 (3)	0.01907 (11)	0.7192 (2)	0.0708 (7)	
H25A	0.534315	−0.010084	0.665401	0.085*	
H25B	0.462154	0.038469	0.713275	0.085*	
C26	0.5819 (4)	−0.00665 (15)	0.8302 (3)	0.1045 (12)	
H26A	0.659852	−0.028957	0.834056	0.157*	
H26B	0.510343	−0.029452	0.843495	0.157*	
H26C	0.598597	0.022160	0.883486	0.157*	
C27	0.7664 (9)	−0.0197 (4)	0.6193 (9)	0.088 (2)	0.596 (10)
H27A	0.777363	−0.044631	0.680528	0.105*	0.596 (10)
H27B	0.682208	−0.027963	0.573177	0.105*	0.596 (10)
C28	0.8758 (9)	−0.0294 (3)	0.5578 (7)	0.125 (3)	0.596 (10)
H28A	0.852852	−0.012733	0.487817	0.187*	0.596 (10)
H28B	0.888376	−0.068523	0.550261	0.187*	0.596 (10)
H28C	0.956043	−0.013087	0.596236	0.187*	0.596 (10)
C27B	0.8116 (14)	−0.0203 (7)	0.6527 (12)	0.082 (3)	0.404 (10)
H27C	0.907171	−0.023151	0.669858	0.099*	0.404 (10)
H27D	0.773839	−0.044287	0.700621	0.099*	0.404 (10)
C28B	0.7591 (16)	−0.0344 (5)	0.5362 (10)	0.123 (4)	0.404 (10)
H28D	0.777508	−0.072623	0.523581	0.185*	0.404 (10)
H28E	0.801120	−0.011260	0.490895	0.185*	0.404 (10)
H28F	0.665400	−0.028341	0.520112	0.185*	0.404 (10)

### Atomic displacement parameters (Å^2^)

**Table:**

	*U*^11^	*U*^22^	*U*^33^	*U*^12^	*U*^13^	*U*^23^
Ag1	0.05433 (12)	0.06584 (14)	0.1134 (2)	0.00078 (9)	0.03772 (12)	−0.00521 (12)
Ag2	0.04237 (10)	0.05675 (11)	0.07309 (13)	−0.00201 (7)	0.02599 (8)	−0.00126 (9)
O1	0.1078 (18)	0.1112 (18)	0.0684 (13)	0.0367 (14)	−0.0053 (12)	−0.0201 (12)
O2	0.0752 (13)	0.0839 (14)	0.0710 (12)	0.0089 (11)	0.0079 (10)	−0.0057 (11)
O3	0.163 (3)	0.108 (2)	0.115 (2)	0.0615 (19)	−0.0098 (19)	−0.0460 (16)
O4	0.0605 (11)	0.1091 (16)	0.0630 (12)	−0.0214 (11)	0.0131 (9)	−0.0019 (11)
O5	0.0779 (16)	0.168 (3)	0.0800 (15)	0.0122 (16)	−0.0001 (13)	0.0222 (16)
O6	0.105 (2)	0.120 (2)	0.142 (2)	−0.0468 (17)	0.0317 (17)	0.0218 (18)
N1	0.0381 (9)	0.0437 (9)	0.0460 (10)	−0.0011 (7)	0.0125 (7)	0.0001 (7)
N2	0.0389 (9)	0.0446 (9)	0.0483 (10)	−0.0020 (7)	0.0138 (7)	−0.0011 (8)
N3	0.0433 (9)	0.0449 (10)	0.0517 (10)	−0.0019 (8)	0.0170 (8)	0.0005 (8)
N4	0.0604 (13)	0.0612 (13)	0.0779 (15)	0.0082 (10)	0.0314 (11)	−0.0059 (11)
N5	0.0711 (15)	0.0812 (16)	0.0682 (15)	0.0140 (13)	0.0150 (12)	−0.0149 (13)
N6	0.0596 (14)	0.0778 (16)	0.0700 (15)	−0.0021 (11)	0.0220 (12)	−0.0061 (12)
C1	0.0392 (10)	0.0417 (11)	0.0489 (12)	−0.0019 (8)	0.0125 (9)	0.0015 (9)
C2	0.0404 (11)	0.0440 (11)	0.0511 (12)	−0.0006 (9)	0.0131 (9)	0.0010 (9)
C3	0.0396 (10)	0.0424 (11)	0.0369 (10)	−0.0036 (8)	0.0081 (8)	0.0008 (8)
C4	0.0483 (12)	0.0527 (13)	0.0467 (12)	−0.0096 (10)	0.0124 (9)	−0.0004 (10)
C5	0.0594 (14)	0.0473 (12)	0.0415 (12)	−0.0128 (10)	0.0035 (10)	0.0030 (9)
C6	0.0647 (15)	0.0406 (11)	0.0427 (12)	−0.0022 (10)	−0.0002 (10)	−0.0009 (9)
C7	0.0505 (12)	0.0447 (12)	0.0465 (12)	0.0031 (9)	0.0092 (10)	−0.0007 (9)
C8	0.0397 (10)	0.0418 (10)	0.0375 (10)	−0.0006 (8)	0.0072 (8)	0.0014 (8)
C9	0.0795 (19)	0.0568 (15)	0.0760 (18)	−0.0250 (14)	0.0152 (15)	0.0048 (13)
C10	0.092 (2)	0.0421 (13)	0.088 (2)	0.0035 (14)	0.0079 (17)	0.0009 (13)
C11	0.0519 (13)	0.0433 (12)	0.0850 (18)	−0.0076 (10)	0.0303 (12)	−0.0016 (11)
C12	0.084 (2)	0.0665 (17)	0.092 (2)	−0.0005 (15)	0.0425 (17)	0.0214 (15)
C13	0.0517 (13)	0.0430 (12)	0.0767 (16)	0.0006 (10)	0.0256 (12)	0.0024 (11)
C14	0.0704 (17)	0.0570 (15)	0.091 (2)	−0.0047 (13)	0.0242 (15)	−0.0164 (14)
C15	0.0548 (13)	0.0485 (12)	0.0611 (14)	−0.0027 (10)	0.0172 (11)	−0.0005 (10)
C16	0.0757 (17)	0.0535 (14)	0.0841 (18)	0.0063 (13)	0.0315 (15)	−0.0099 (13)
C17	0.0448 (12)	0.0623 (14)	0.0462 (12)	0.0015 (10)	0.0155 (9)	−0.0012 (10)
C18	0.0415 (12)	0.0792 (18)	0.0498 (13)	−0.0044 (11)	0.0169 (10)	0.0004 (12)
C19	0.0478 (12)	0.0741 (16)	0.0376 (11)	−0.0157 (11)	0.0075 (9)	0.0038 (11)
C20	0.0510 (12)	0.0516 (13)	0.0418 (11)	−0.0075 (10)	0.0059 (9)	0.0057 (9)
C21	0.0449 (11)	0.0492 (12)	0.0514 (13)	−0.0006 (9)	0.0159 (10)	0.0003 (10)
C22	0.0390 (10)	0.0485 (12)	0.0434 (11)	−0.0017 (8)	0.0127 (8)	0.0012 (9)
C23	0.0617 (16)	0.093 (2)	0.0579 (15)	−0.0292 (15)	0.0132 (12)	0.0092 (14)
C24	0.0794 (19)	0.0534 (15)	0.0748 (18)	−0.0118 (13)	0.0093 (15)	0.0089 (13)
C25	0.0675 (17)	0.0489 (14)	0.101 (2)	−0.0106 (12)	0.0276 (15)	−0.0025 (14)
C26	0.111 (3)	0.086 (2)	0.128 (3)	0.000 (2)	0.051 (2)	0.033 (2)
C27	0.093 (5)	0.059 (3)	0.116 (6)	0.014 (4)	0.030 (4)	−0.026 (4)
C28	0.095 (5)	0.105 (5)	0.170 (6)	0.020 (4)	0.018 (5)	−0.073 (4)
C27B	0.081 (6)	0.070 (4)	0.096 (6)	−0.006 (5)	0.018 (5)	−0.015 (5)
C28B	0.148 (9)	0.094 (6)	0.122 (7)	0.016 (6)	0.011 (7)	−0.039 (6)

### Geometric parameters (Å, º)

**Table:**

Ag1—O1	2.498 (2)	C12—H12C	0.9600
Ag1—O2	2.512 (2)	C13—H13A	0.9700
Ag1—O4^i^	2.3195 (19)	C13—H13B	0.9700
Ag1—N1	2.2600 (17)	C13—C14	1.512 (4)
Ag2—O4	2.5956 (19)	C14—H14A	0.9600
Ag2—N2	2.2492 (17)	C14—H14B	0.9600
Ag2—N3	2.2552 (17)	C14—H14C	0.9600
O1—N5	1.251 (3)	C15—C16	1.427 (4)
O2—N5	1.223 (3)	C15—C25	1.506 (3)
O3—N5	1.229 (3)	C16—C27	1.550 (10)
O4—N6	1.257 (3)	C16—C27B	1.563 (17)
O5—N6	1.215 (3)	C17—C18	1.410 (3)
O6—N6	1.220 (3)	C17—C22	1.403 (3)
N1—C1	1.320 (3)	C18—H18	0.9300
N1—C8	1.373 (3)	C18—C19	1.359 (4)
N2—C2	1.328 (3)	C19—C20	1.430 (3)
N2—C3	1.371 (3)	C19—C23	1.510 (3)
N3—C15	1.323 (3)	C20—C21	1.376 (3)
N3—C22	1.378 (3)	C20—C24	1.503 (3)
N4—C16	1.306 (3)	C21—H21	0.9300
N4—C17	1.366 (3)	C21—C22	1.407 (3)
C1—C2	1.430 (3)	C23—H23A	0.9600
C1—C11	1.505 (3)	C23—H23B	0.9600
C2—C13	1.507 (3)	C23—H23C	0.9600
C3—C4	1.408 (3)	C24—H24A	0.9600
C3—C8	1.403 (3)	C24—H24B	0.9600
C4—H4	0.9300	C24—H24C	0.9600
C4—C5	1.375 (3)	C25—H25A	0.9700
C5—C6	1.421 (3)	C25—H25B	0.9700
C5—C9	1.512 (3)	C25—C26	1.515 (4)
C6—C7	1.368 (3)	C26—H26A	0.9600
C6—C10	1.512 (3)	C26—H26B	0.9600
C7—H7	0.9300	C26—H26C	0.9600
C7—C8	1.408 (3)	C27—H27A	0.9700
C9—H9A	0.9600	C27—H27B	0.9700
C9—H9B	0.9600	C27—C28	1.504 (13)
C9—H9C	0.9600	C28—H28A	0.9600
C10—H10A	0.9600	C28—H28B	0.9600
C10—H10B	0.9600	C28—H28C	0.9600
C10—H10C	0.9600	C27B—H27C	0.9700
C11—H11A	0.9700	C27B—H27D	0.9700
C11—H11B	0.9700	C27B—C28B	1.508 (19)
C11—C12	1.522 (4)	C28B—H28D	0.9600
C12—H12A	0.9600	C28B—H28E	0.9600
C12—H12B	0.9600	C28B—H28F	0.9600
			
O1—Ag1—O2	50.25 (7)	C14—C13—H13A	109.0
O4^i^—Ag1—O1	95.46 (9)	C14—C13—H13B	109.0
O4^i^—Ag1—O2	116.53 (7)	C13—C14—H14A	109.5
N1—Ag1—O1	125.80 (8)	C13—C14—H14B	109.5
N1—Ag1—O2	113.10 (7)	C13—C14—H14C	109.5
N1—Ag1—O4^i^	129.00 (7)	H14A—C14—H14B	109.5
N2—Ag2—O4	101.47 (6)	H14A—C14—H14C	109.5
N2—Ag2—N3	173.50 (6)	H14B—C14—H14C	109.5
N3—Ag2—O4	80.37 (7)	N3—C15—C16	120.8 (2)
N5—O1—Ag1	95.48 (17)	N3—C15—C25	117.0 (2)
N5—O2—Ag1	95.56 (16)	C16—C15—C25	122.2 (2)
Ag1^ii^—O4—Ag2	138.85 (9)	N4—C16—C15	122.2 (2)
N6—O4—Ag1^ii^	107.43 (16)	N4—C16—C27	120.0 (4)
N6—O4—Ag2	112.28 (16)	N4—C16—C27B	110.5 (6)
C1—N1—Ag1	122.18 (13)	C15—C16—C27	117.2 (4)
C1—N1—C8	118.57 (18)	C15—C16—C27B	126.3 (6)
C8—N1—Ag1	119.25 (14)	N4—C17—C18	119.7 (2)
C2—N2—Ag2	122.50 (14)	N4—C17—C22	121.1 (2)
C2—N2—C3	118.51 (18)	C22—C17—C18	119.2 (2)
C3—N2—Ag2	118.47 (13)	C17—C18—H18	119.2
C15—N3—Ag2	121.44 (15)	C19—C18—C17	121.6 (2)
C15—N3—C22	118.08 (19)	C19—C18—H18	119.2
C22—N3—Ag2	120.23 (14)	C18—C19—C20	119.4 (2)
C16—N4—C17	117.8 (2)	C18—C19—C23	120.2 (2)
O2—N5—O1	118.7 (2)	C20—C19—C23	120.3 (2)
O2—N5—O3	119.5 (3)	C19—C20—C24	120.6 (2)
O3—N5—O1	121.8 (3)	C21—C20—C19	119.6 (2)
O5—N6—O4	116.6 (2)	C21—C20—C24	119.8 (2)
O5—N6—O6	124.3 (3)	C20—C21—H21	119.5
O6—N6—O4	118.9 (3)	C20—C21—C22	121.0 (2)
N1—C1—C2	121.28 (18)	C22—C21—H21	119.5
N1—C1—C11	116.77 (19)	N3—C22—C17	120.0 (2)
C2—C1—C11	121.92 (19)	N3—C22—C21	120.81 (19)
N2—C2—C1	120.71 (19)	C17—C22—C21	119.1 (2)
N2—C2—C13	117.43 (19)	C19—C23—H23A	109.5
C1—C2—C13	121.85 (19)	C19—C23—H23B	109.5
N2—C3—C4	120.30 (19)	C19—C23—H23C	109.5
N2—C3—C8	120.60 (18)	H23A—C23—H23B	109.5
C8—C3—C4	119.09 (19)	H23A—C23—H23C	109.5
C3—C4—H4	119.3	H23B—C23—H23C	109.5
C5—C4—C3	121.4 (2)	C20—C24—H24A	109.5
C5—C4—H4	119.3	C20—C24—H24B	109.5
C4—C5—C6	119.1 (2)	C20—C24—H24C	109.5
C4—C5—C9	119.7 (2)	H24A—C24—H24B	109.5
C6—C5—C9	121.2 (2)	H24A—C24—H24C	109.5
C5—C6—C10	120.5 (2)	H24B—C24—H24C	109.5
C7—C6—C5	120.0 (2)	C15—C25—H25A	109.2
C7—C6—C10	119.5 (2)	C15—C25—H25B	109.2
C6—C7—H7	119.4	C15—C25—C26	112.0 (3)
C6—C7—C8	121.2 (2)	H25A—C25—H25B	107.9
C8—C7—H7	119.4	C26—C25—H25A	109.2
N1—C8—C3	120.29 (18)	C26—C25—H25B	109.2
N1—C8—C7	120.59 (19)	C25—C26—H26A	109.5
C3—C8—C7	119.11 (19)	C25—C26—H26B	109.5
C5—C9—H9A	109.5	C25—C26—H26C	109.5
C5—C9—H9B	109.5	H26A—C26—H26B	109.5
C5—C9—H9C	109.5	H26A—C26—H26C	109.5
H9A—C9—H9B	109.5	H26B—C26—H26C	109.5
H9A—C9—H9C	109.5	C16—C27—H27A	109.2
H9B—C9—H9C	109.5	C16—C27—H27B	109.2
C6—C10—H10A	109.5	H27A—C27—H27B	107.9
C6—C10—H10B	109.5	C28—C27—C16	111.8 (7)
C6—C10—H10C	109.5	C28—C27—H27A	109.2
H10A—C10—H10B	109.5	C28—C27—H27B	109.2
H10A—C10—H10C	109.5	C27—C28—H28A	109.5
H10B—C10—H10C	109.5	C27—C28—H28B	109.5
C1—C11—H11A	109.2	C27—C28—H28C	109.5
C1—C11—H11B	109.2	H28A—C28—H28B	109.5
C1—C11—C12	111.9 (2)	H28A—C28—H28C	109.5
H11A—C11—H11B	107.9	H28B—C28—H28C	109.5
C12—C11—H11A	109.2	C16—C27B—H27C	111.3
C12—C11—H11B	109.2	C16—C27B—H27D	111.3
C11—C12—H12A	109.5	H27C—C27B—H27D	109.2
C11—C12—H12B	109.5	C28B—C27B—C16	102.2 (10)
C11—C12—H12C	109.5	C28B—C27B—H27C	111.3
H12A—C12—H12B	109.5	C28B—C27B—H27D	111.3
H12A—C12—H12C	109.5	C27B—C28B—H28D	109.5
H12B—C12—H12C	109.5	C27B—C28B—H28E	109.5
C2—C13—H13A	109.0	C27B—C28B—H28F	109.5
C2—C13—H13B	109.0	H28D—C28B—H28E	109.5
C2—C13—C14	113.0 (2)	H28D—C28B—H28F	109.5
H13A—C13—H13B	107.8	H28E—C28B—H28F	109.5
			
Ag1—O1—N5—O2	2.3 (3)	C3—C4—C5—C9	178.3 (2)
Ag1—O1—N5—O3	−177.4 (3)	C4—C3—C8—N1	−178.48 (18)
Ag1—O2—N5—O1	−2.2 (3)	C4—C3—C8—C7	2.4 (3)
Ag1—O2—N5—O3	177.4 (3)	C4—C5—C6—C7	3.6 (3)
Ag1^ii^—O4—N6—O5	5.1 (3)	C4—C5—C6—C10	−174.5 (2)
Ag1^ii^—O4—N6—O6	−178.1 (2)	C5—C6—C7—C8	−3.1 (3)
Ag1—N1—C1—C2	−179.96 (15)	C6—C7—C8—N1	−179.0 (2)
Ag1—N1—C1—C11	−2.1 (3)	C6—C7—C8—C3	0.1 (3)
Ag1—N1—C8—C3	−179.52 (14)	C8—N1—C1—C2	0.2 (3)
Ag1—N1—C8—C7	−0.4 (3)	C8—N1—C1—C11	178.1 (2)
Ag2—O4—N6—O5	174.1 (2)	C8—C3—C4—C5	−1.9 (3)
Ag2—O4—N6—O6	−9.1 (3)	C9—C5—C6—C7	−175.8 (2)
Ag2—N2—C2—C1	−169.46 (15)	C9—C5—C6—C10	6.1 (3)
Ag2—N2—C2—C13	9.8 (3)	C10—C6—C7—C8	175.0 (2)
Ag2—N2—C3—C4	−10.8 (3)	C11—C1—C2—N2	−179.3 (2)
Ag2—N2—C3—C8	170.33 (14)	C11—C1—C2—C13	1.4 (3)
Ag2—N3—C15—C16	−173.9 (2)	C15—N3—C22—C17	−1.4 (3)
Ag2—N3—C15—C25	4.2 (3)	C15—N3—C22—C21	176.9 (2)
Ag2—N3—C22—C17	172.84 (16)	C15—C16—C27—C28	166.0 (6)
Ag2—N3—C22—C21	−8.8 (3)	C15—C16—C27B—C28B	97.3 (11)
N1—C1—C2—N2	−1.5 (3)	C16—N4—C17—C18	−176.9 (2)
N1—C1—C2—C13	179.2 (2)	C16—N4—C17—C22	0.5 (4)
N1—C1—C11—C12	−88.0 (3)	C16—C15—C25—C26	83.2 (3)
N2—C2—C13—C14	−92.9 (3)	C17—N4—C16—C15	−1.7 (4)
N2—C3—C4—C5	179.20 (19)	C17—N4—C16—C27	168.7 (5)
N2—C3—C8—N1	0.4 (3)	C17—N4—C16—C27B	−170.5 (6)
N2—C3—C8—C7	−178.69 (19)	C17—C18—C19—C20	1.7 (3)
N3—C15—C16—N4	1.3 (4)	C17—C18—C19—C23	−176.2 (2)
N3—C15—C16—C27	−169.3 (5)	C18—C17—C22—N3	178.5 (2)
N3—C15—C16—C27B	168.4 (6)	C18—C17—C22—C21	0.1 (3)
N3—C15—C25—C26	−94.9 (3)	C18—C19—C20—C21	0.1 (3)
N4—C16—C27—C28	−4.8 (10)	C18—C19—C20—C24	−178.6 (2)
N4—C16—C27B—C28B	−94.4 (11)	C19—C20—C21—C22	−1.7 (3)
N4—C17—C18—C19	175.7 (2)	C20—C21—C22—N3	−176.8 (2)
N4—C17—C22—N3	1.1 (3)	C20—C21—C22—C17	1.6 (3)
N4—C17—C22—C21	−177.3 (2)	C22—N3—C15—C16	0.3 (4)
C1—N1—C8—C3	0.3 (3)	C22—N3—C15—C25	178.4 (2)
C1—N1—C8—C7	179.36 (19)	C22—C17—C18—C19	−1.8 (3)
C1—C2—C13—C14	86.4 (3)	C23—C19—C20—C21	177.9 (2)
C2—N2—C3—C4	177.23 (19)	C23—C19—C20—C24	−0.7 (3)
C2—N2—C3—C8	−1.6 (3)	C24—C20—C21—C22	177.0 (2)
C2—C1—C11—C12	89.8 (3)	C25—C15—C16—N4	−176.7 (3)
C3—N2—C2—C1	2.2 (3)	C25—C15—C16—C27	12.7 (6)
C3—N2—C2—C13	−178.54 (19)	C25—C15—C16—C27B	−9.7 (7)
C3—C4—C5—C6	−1.1 (3)		

Symmetry codes: (i) *x*−1, *y*, *z*; (ii) *x*+1, *y*, *z*.
